# Cross-sectional associations of Kidney deficiency, Phlegm-stasis, and Heat-toxin syndrome patterns with disease severity in Alzheimer’s disease: an exploratory study

**DOI:** 10.3389/fnagi.2026.1763273

**Published:** 2026-10-16

**Authors:** Limin Zhang, Hongmei An, Minrui Ding, Xiaofei Yu, Feng Yan, Chao Gu, Xia Li

**Affiliations:** 1 Department of Geriatric Psychiatry, Shanghai Mental Health Center, Shanghai Jiao Tong University School of Medicine, Shanghai, China; 2 Department of Neurology, Longhua Hospital, Shanghai University of Traditional Chinese Medicine, Shanghai, China; 3 Department of Science and Technology, Longhua Hospital, Shanghai University of Traditional Chinese Medicine, Shanghai, China; 4 Department of Special Needs, Longhua Hospital, Shanghai University of Traditional Chinese Medicine, Shanghai, China

**Keywords:** Alzheimer’s disease, biomarkers, cross-sectional analysis, exploratory discriminatory performance, Traditional Chinese Medicine syndromes

## Abstract

**Background:**

Current Alzheimer’s diagnostics lack integration with Traditional Chinese Medicine’s syndrome differentiation framework, making it difficult to fully capture the clinical heterogeneity and multidimensional nature of the disease.

**Methods:**

This cross-sectional study analyzed 236 AD patients stratified by CDR into early-stage (*n* = 127), moderate (*n* = 66), and severe (*n* = 43) groups. Using the SHA-03-W1 diagnostic instrument and Tian Jinzhou Scale, patients were classified into three TCM syndrome patterns: Kidney essence deficiency (*n* = 87), Phlegm-stasis obstructing the orifices (*n* = 93), and Heat-toxin obstructing the collaterals (*n* = 56). Data collected included demographics, cognitive scores (MMSE, MoCA), activities of daily living (ADL), and plasma biomarkers (GFAP, NfL, p-tau181, p-tau217, Aβ42/40). Statistical analyses involved intergroup comparisons (Chi-square and Kruskal-Wallis tests), Spearman correlation analysis between dementia severity/TCM patterns and clinical measures, logistic regression to examine factors associated with disease severity, and exploratory ROC analysis to describe discriminatory performance within this development sample.

**Results:**

Across the three CDR-defined severity groups (*n* = 236), MMSE, MoCA, ADL and estimated symptom duration differed significantly (*p* < 0.05, *q* < 0.05). Dementia severity correlated strongly with MMSE (*r* = −0.779), MoCA (*r* = −0.785) and ADL (*r* = 0.742) (*p* < 0.001), and only weakly with estimated symptom duration (*r* = 0.384, *p* < 0.001). TCM syndrome distribution patterns differed across severity stages, with kidney essence deficiency being most prevalent in the early-stage group (48%), Phlegm-stasis in the moderate group (54.5%), and Heat-toxin in the severe group (60.5%). In multivariable multinomial logistic regression (reference: early-stage AD), MMSE and ADL scores were associated with AD severity (MMSE: moderate vs. early, OR = 0.844, 95% CI 0.774–0.921; severe vs. early, OR = 0.485, 95% CI 0.351–0.671; ADL: moderate vs. early, OR = 1.128, 95% CI 1.065–1.196; severe vs. early, OR = 1.280, 95% CI 1.150–1.425; all *p* < 0.001). Among TCM syndrome elements, Heat-toxin was associated with both moderate and severe AD (Moderate vs. Early-stage: OR = 1.177, 95% CI 1.046–1.324, *p* = 0.007; Severe vs. Early-stage: OR = 1.498, 95% CI 1.205–1.861, *p* < 0.001) and Phlegm-stasis with moderate AD only (OR = 1.198, 95% CI 1.079–1.329, *p* = 0.001). In an exploratory ROC analysis, single indicators showed modest discriminatory performance for Heat-toxin syndrome, with CDR (AUC = 0.709) > ADL (AUC = 0.690) > MMSE (AUC = 0.683), whereas AUCs for Kidney deficiency (≤0.633) and Phlegm-stasis (≤0.581) were close to chance. In a sensitivity analysis excluding participants with CDR 0.5 (*n* = 186), the cognitive, functional and Heat-toxin associations were essentially unchanged, but the Phlegm-stasis score was associated with both moderate and severe AD, with comparable effect sizes (Moderate vs. Early-stage: OR = 1.26, 95% CI 1.12–1.42, *p* < 0.001; Severe vs. Early-stage: OR = 1.25, 95% CI 1.02–1.54, *p =* 0.032), indicating that its restriction to moderate AD was not robust.

**Discussion:**

In this cross-sectional single-center sample, TCM syndrome distributions and scores differed across concurrent CDR-defined severity groups, with kidney essence deficiency predominating in early-stage AD (48.0%), Phlegm-stasis in moderate AD (54.5%), and Heat-toxin in severe AD (60.5%). Heat-toxin scores were associated with greater concurrent cognitive and functional impairment, whereas plasma biomarkers showed limited and inconsistent associations. These findings do not establish temporal syndrome transitions or validated diagnostic utility outside this development sample, but provide hypothesis-generating evidence for future prospective investigations.

## 1 Introduction

Traditional Chinese Medicine (TCM) has a long-standing history in conceptualizing Alzheimer’s disease (AD), which it categorizes under conditions such as “dementia” and “amnesia,” and has accordingly developed a distinctive system of syndrome differentiation and treatment (Li et al., 2021). Historically, most TCM scholars have regarded “Kidney deficiency” as the root cause of the disease. As recorded in Yixue Xinzhu, “the kidney governs intelligence; Kidney deficiency leads to insufficiency of intelligence,” suggesting that depletion of kidney essence and failure to nourish the marrow sea underlie subjective cognitive decline (SCD) (Liu et al., 2014). As the condition progresses, the underlying deficiency is thought to disturb fluid metabolism and impede the free flow of qi and blood, giving rise to patterns described as “phlegm-turbidity clouding the orifices” and “static blood obstructing the collaterals.” Shishi Milu emphasizes that “where phlegm is most exuberant, dementia is most profound,” highlighting the role of phlegm-turbidity in cognitive impairment (Liu et al., 2025). Similarly, Yilin Gaicuo observes that “static blood makes one prone to forgetfulness,” pointing to the involvement of blood stasis in memory decline. In the advanced stages, prolonged accumulation of phlegm and stasis is theorized to generate heat and transform into toxin, manifesting as complex patterns such as “internal exuberance of Heat-toxin” and “bowel stagnation with turbidity retention,” and potentially progressing to critical states including “loss of mental control” and “collapse of healthy qi” (Zhang et al., 2023). Synthesizing these classical accounts, the pathogenesis of AD in TCM can be conceptualized as Kidney deficiency as the root, with phlegm and stasis as the secondary manifestations; in the later stages, the enduring accumulation of phlegm and stasis generates heat and toxin, culminating in a pathological state of “toxin damaging the brain collaterals” (Dong et al., 2025). This hypothesized sequence—from Kidney deficiency to Phlegm-stasis to toxin injury—offers a holistic framework for understanding the course of AD.

The pathogenesis of AD is complex and remains incompletely understood. Recent advances in single-molecule array technology have substantially improved the sensitivity and reliability of detecting low-abundance peripheral blood biomarkers, including p-tau217, GFAP, and NfL, opening new avenues for early diagnosis and disease monitoring (Pacoova Dal Maschio et al., 2025). These biomarkers reflect multiple pathological dimensions—cerebral Aβ deposition, tau pathology, and axonal injury—thereby moving AD diagnosis beyond purely clinical assessment toward a biologically grounded framework. However, despite the promising performance of blood-based biomarkers in distinguishing AD patients from healthy controls, their relationship with clinical heterogeneity remains poorly characterized. Some studies have reported associations between p-tau217 and the rate of SCD, whereas others suggest that its levels plateau in the moderate-to-severe stages. Similarly, GFAP—a marker of astroglial activation or injury—has shown inconsistent trajectories across AD stages (Cui et al., 2026). These discrepant findings suggest that a single biomarker is unlikely to capture the full complexity of AD progression, underscoring the need for multidimensional assessment approaches.

In recent years, efforts to objectify and standardize TCM syndrome differentiation and treatment evaluation in AD have gained momentum, with information-based four-diagnostic technology playing an increasingly important role. The SHA-03-W1 four-diagnostic instrument, for instance, captures objective data on tongue appearance, pulse waveforms, and facial complexion, and integrates image processing with data-mining techniques to enable quantitative analysis of TCM syndromes (Ren et al., 2020). Our research group previously examined the distribution of TCM syndrome types across populations with SCD, early-stage cognitive impairment (MCI), and AD. The findings showed that the frequency of syndrome types in AD patients, in descending order, was kidney essence deficiency, phlegm-turbidity obstructing the orifices, blood stasis obstructing the collaterals, internal exuberance of Heat-toxin, and bowel stagnation with turbidity retention. These results provided a preliminary description of syndrome distributions across different clinical stages and laid the groundwork for further investigations into the patterning of TCM syndromes across varying degrees of dementia severity (Zhang et al., 2024).

While Western medicine offers distinct advantages in understanding and intervening in AD—for example, peripheral blood biomarkers such as Aβ42 and p-tau have demonstrated significant utility in early diagnosis and disease monitoring (Arslan et al., 2024)—few studies have attempted to construct a diagnostic or assessment framework that integrates the strengths of both medical systems. Moreover, the relationship between AD and the TCM syndrome pattern sequence of “Kidney deficiency → Phlegm-stasis → toxin injury” has yet to be thoroughly investigated. To address this gap, the present study employs a cross-sectional design in which Western clinical cognitive scales (MMSE, MoCA, ADL, CDR) define disease stages (early-stage, moderate, and severe AD) as one dimension, while TCM syndrome patterns (kidney essence deficiency, Phlegm-stasis obstructing the orifices, Heat-toxin obstructing the collaterals), identified through quantitative data from the SHA-03-W1 instrument, constitute the other. The study will explore the concurrent associations between TCM syndrome types and AD severity stages. Besides, it will characterize how the weighting of TCM syndrome elements varies across severity levels. Moreover, it will provide empirical evidence that may refine the hypothesized “Kidney deficiency → Phlegm-stasis → Heat-toxin” distribution pattern within a cross-sectional AD sample.

## 2 Materials and methods

### 2.1 Research subjects

#### 2.1.1 Source of cases

The research cases were collected from patients diagnosed with AD at the Shanghai Mental Health Center between February 2025 and August 2025. This cohort includes both inpatient and outpatient individuals.

#### 2.1.2 Diagnostic criteria

(1) Diagnostic criteria for AD: Patients must meet the highly probable AD diagnostic criteria established in 2011 by the National Institute on Aging (NIA) and the Alzheimer’s Association (AA) (McKhann et al., 2011).

(2) The diagnosis of TCM syndromes for AD primarily refers to Professor Tian Jinzhou’s Dementia Syndrome Differentiation Scale. A score of ≥7 points establish the diagnosis of a specific syndrome. The severity is graded as follows: a score of 7–14 points indicates a early-stage degree; a score of 15–22 points indicates a moderate degree; and a score of 23–30 points indicates a severe degree (Tian et al., 2000, 2021).

#### 2.1.3 Inclusion criteria

(1) Age 40–90 years, with no gender restrictions.

(2) Meets the diagnostic criteria for dementia resulting from AD as defined by the 2011 NIA-AA guidelines.

(3) Participants must have a stable and reliable caregiver or attendant who engages in sufficient interaction and communication with the patient to provide valuable information for assessments, including ADL and the TCM syndrome classification scale.

(4) Comprehensive personal and medical history information must be accessible for statistical analysis.

(5) Informed consent shall be obtained from the subject or their legal guardian. early-stage AD patients with capacity for informed consent shall sign the consent form themselves. Moderate-to-severe AD patients assessed as lacking such capacity shall have the consent form signed by their legal guardian on their behalf. Both approaches are considered valid for enrollment.

#### 2.1.4 Exclusion criteria

(1) Dementia may result from various etiologies including vascular dementia, neurodegenerative disorders (Parkinson’s, Huntington’s, Lewy body), CNS infections (HIV, syphilis, prion diseases), traumatic brain injury, metabolic disturbances (endocrine disorders, nutritional deficiencies), toxic exposures, systemic illnesses, and intracranial space-occupying lesions.

(2) Individuals with co-occurring severe depression are identified by a Geriatric Depression Scale (GDS) score of 20 or higher.

(3) Congenital intellectual disability.

(4) Potential participants were excluded if investigators determined they were unable to comply with study requirements.

### 2.2 Research methods

#### 2.2.1 Grouping methods

Patients were categorized by dementia severity using the Clinical Dementia Rating (CDR) scale: early-stage (CDR = 0.5–1.0), moderate (CDR = 2.0), and severe (CDR = 3.0) (Vakkila and Jehkonen, 2023). TCM syndrome differentiation identified three patterns: kidney essence deficiency, Phlegm-stasis obstructing orifices, and Heat-toxin obstructing collaterals. Syndrome diagnosis required a total score ≥7 points based on SHA-03-W1 assessment, with the highest-scoring syndrome designated as primary and others as secondary (Figure 1).

**FIGURE 1:**
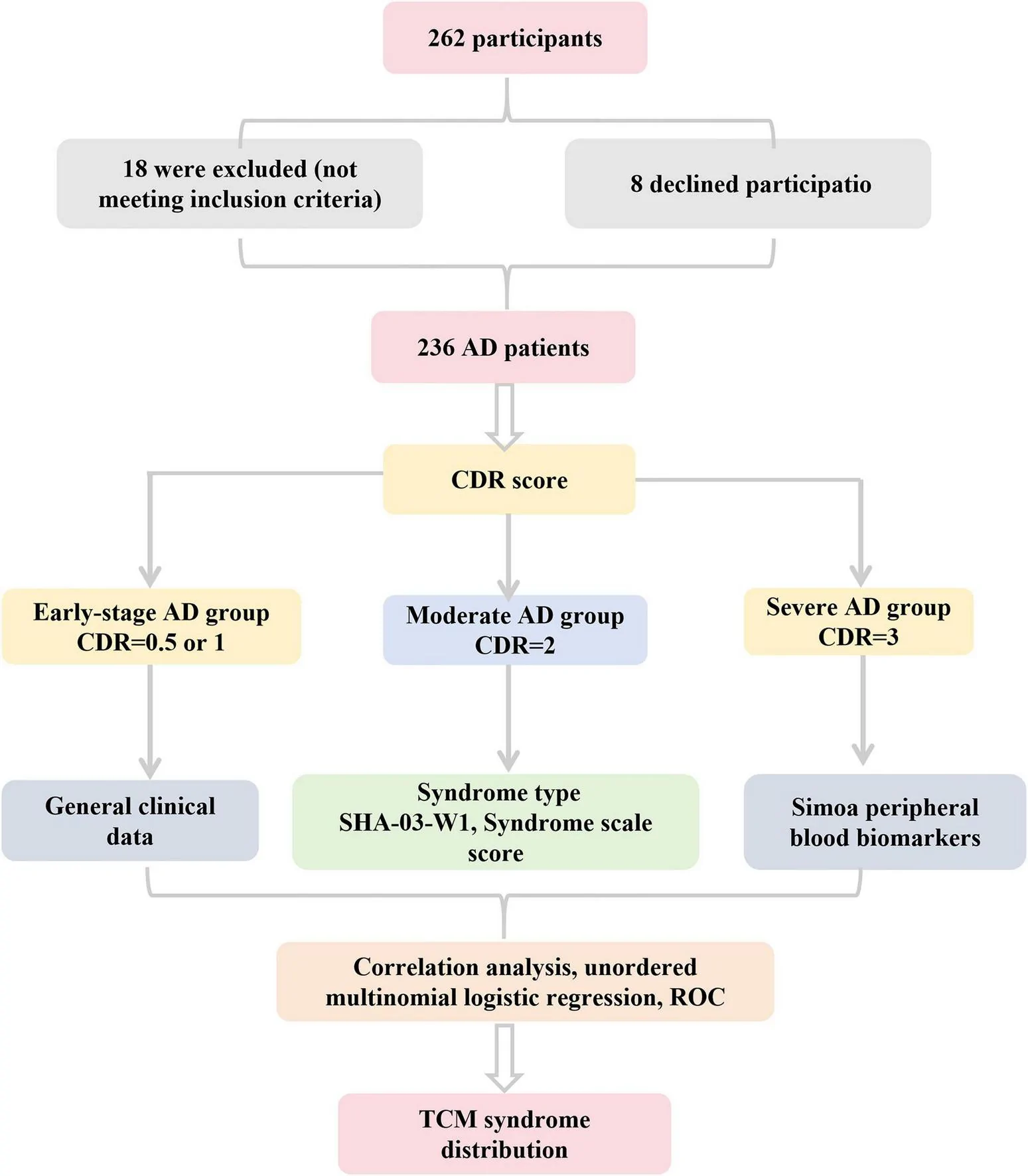
Schematic diagram.

#### 2.2.2 General information

Cognitive and functional assessments were conducted using standardized instruments administered by trained neuropsychology practitioners. The ADL scale (Zhang and He, 1998) comprises 14 items (physical self-maintenance and instrumental subscales), scored 1–4 per item, total range 14–56, with higher scores indicating greater impairment. The MMSE Chinese version (Katzman et al., 1988) includes 30 items (range 0–30); education-based cutoffs were applied (≤17 for illiterate, ≤20 for primary, ≤24 for secondary+). The MoCA Mandarin version (Jack et al., 2018; Scheltens et al., 2021) (range 0–30) included a 1-point education adjustment for ≤12 years of schooling. “Estimated symptom duration” was defined as the interval from first SCD (per participant/caregiver recall) to enrollment.

#### 2.2.3 Standardized data acquisition using the TCM four diagnostic instruments

The SHA-03-W1 diagnostic platform (Tianjin National Health Technology Co., Ltd.; software version 2.38.0) integrates a tongue imaging module (GMSX001-W1), an automated pulse diagnosis module (GMMX002-W1), and intelligent analysis software, and was employed for standardized acquisition and analysis of tongue, facial, and pulse data. Tongue images were captured under LED-backlit conditions and quantitatively analyzed for color, texture, morphology, moisture, and pathological features (e.g., tooth marks, petechiae, coating distribution). Facial diagnostics evaluated complexion characteristics including hue, lip/period orbital color, moisture, and luminosity. Pulse diagnostics employed a pressure-sensing array to capture radial artery waveforms at superficial, intermediate, and deep levels, extracting parameters for pulse position, rhythm, intensity, and pattern classification. Constitution types were determined per the CACM standard ZYYXH-T157-2009 based on integrated questionnaire and diagnostic data. All analyses were performed using the instrument’s proprietary software to support syndrome differentiation (Figure 2).

**FIGURE 2:**
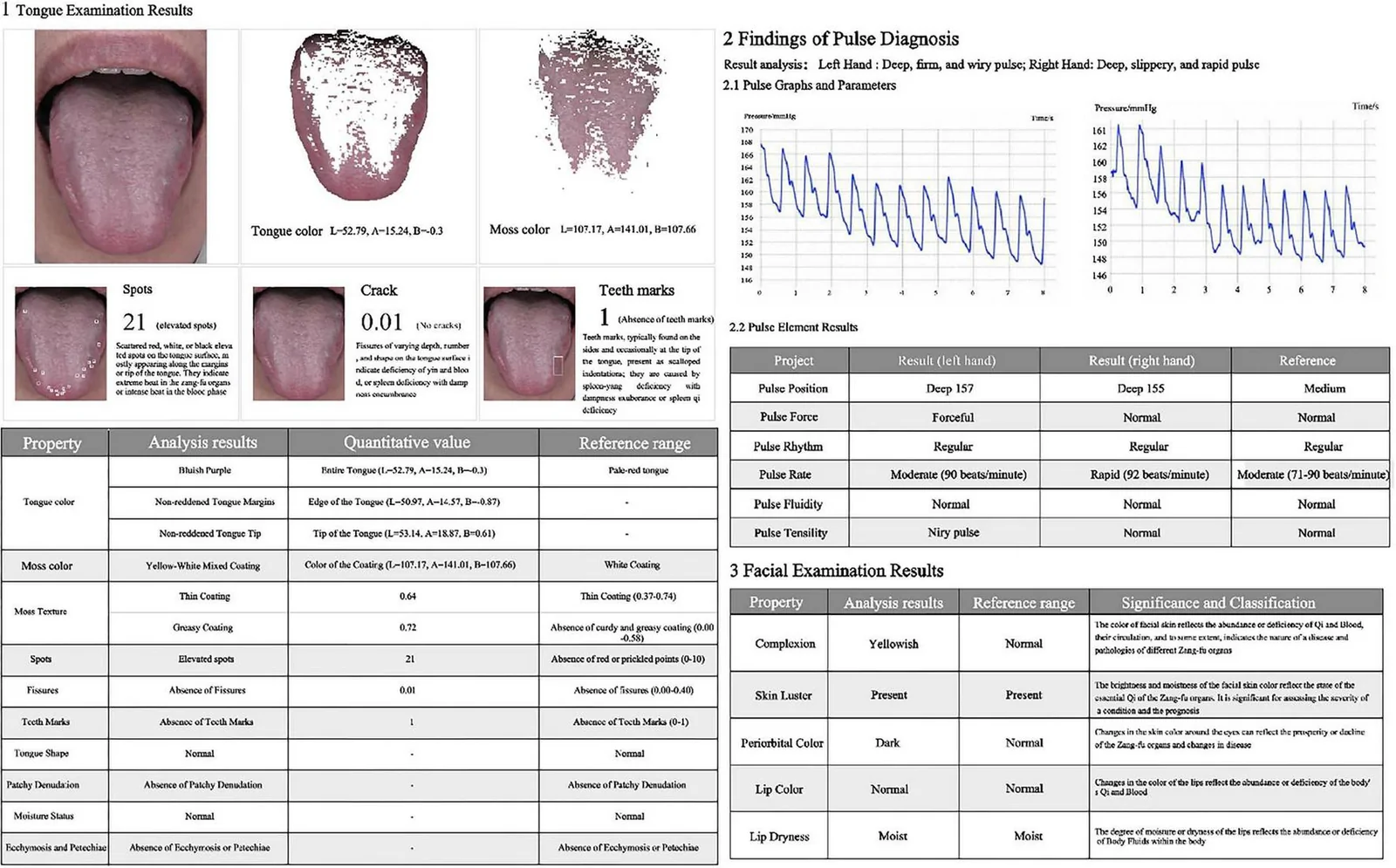
Data collection and analysis of the Traditional Chinese Medicine (TCM) four diagnostic instrument.

#### 2.2.4 Simoa detection of peripheral blood biomarkers

Plasma levels of glial fibrillary acidic protein (GFAP), phosphorylated tau-217 (p-tau217), phosphorylated tau-181 (p-tau181), neurofilament light chain (NfL), β-amyloid 1−40 (Aβ_1–40_), and β-amyloid 1−42 (Aβ_1–42_) were quantified using the AST-Dx90 fully automated single-molecule fluorescence immunoassay analyzer (Suzhou ASTRABIO Co., Ltd., Suzhou, China). All samples were analyzed in a single analytical batch using manufacturer-supplied reagents and standardized operating procedures.

For sample preprocessing, plasma samples stored at −80 °C were thawed at 2 °C–8 °C. After complete thawing, samples were centrifuged at 13,000 × *g* for 10 min at 4 °C, and the clear supernatant was collected for analysis. Undiluted plasma was directly used for biomarker measurements. During instrument preparation, the analyzer was initialized according to the manufacturer’s standard operating procedures. For reagent loading, calibration, and quality control, assay reagents—including the Aβ_1–40_ Assay Kit (Catalog No. R14010), Aβ_1–42_ Assay Kit (Catalog No. R14020), Phosphorylated Tau-181 Protein Assay Kit (Catalog No. R14030), Neurofilament Light Chain Protein Assay Kit (Catalog No. R14040), Phosphorylated Tau-217 Protein Assay Kit (Catalog No. R14050), and Glial Fibrillary Acidic Protein Assay Kit (Catalog No. R14060; all from Suzhou ASTRABIO Co., Ltd., Suzhou, China)—were mixed, equilibrated, and loaded onto the reagent carousel. Calibration and quality-control procedures were performed for each biomarker before sample measurements.

Following sample loading, biomarker concentrations were automatically measured by the analyzer and exported via the instrument software, with results reported in pg/mL. For descriptive interpretation, manufacturer-provided reference ranges were applied where available: plasma NfL < 30 pg/mL, p-tau181 < 4.8 pg/mL, and p-tau217 < 5.9 pg/mL were considered within the reference range, whereas GFAP concentrations of 30–100 pg/mL were regarded as potentially indicative of neurodegenerative changes. No manufacturer-provided cut-off values were available for Aβ_1–40_, Aβ_1–42_, or the Aβ42/Aβ40 ratio.

#### 2.2.5 Statistical methods

Statistical analyses were performed using SPSS 25.0 and R 4.4.2 software. Categorical variables are presented as frequencies (percentages) and compared using the chi-square test. Quantitative variables were assessed for normality; non-normally distributed data are reported as median (interquartile range) and compared across groups with the Kruskal-Wallis test. Spearman’s rank correlation was employed for correlation analysis. A two-sided *p* < 0.05 was considered statistically significant. Group comparisons and correlation analyses were designated as primary analyses, while logistic regression and ROC analyses were considered exploratory and hypothesis-generating, with their results requiring cautious interpretation and prospective validation.

Missing data. Data completeness was high: All variables except the depression score (GDS-30) had <1.3% missing values (range 0.0%–1.3%, median 0.0%; 0.4% of all data points), and no missing values were present for any plasma biomarker; missingness was sporadic, with only one participant missing more than one variable. Missing data were not imputed: group comparisons and correlations used an available-case basis (denominators vary slightly across variables).

Multiple testing: For the comparison across the three TCM syndrome groups, the family of tests comprised all 20 variables. The 12 continuous variables (age, years of education, MMSE, MoCA, ADL, estimated symptom duration, GFAP, NfL, p-tau181, p-tau217, Aβ42/40, and depression score) were compared using the Kruskal–Wallis test, and the eight categorical variables (sex, smoking, alcohol consumption, hypertension, heart disease, diabetes, hyperlipidemia, and family history of dementia) using the χ^2^ test. The Benjamini–Hochberg procedure was applied within this family of 20 tests. For the comparison across the three Western-medicine diagnostic groups, the family of tests comprised the same 20 variables (12 continuous and eight categorical), analyzed using the same tests as described above. The Benjamini–Hochberg procedure was applied within this family of 20 tests to control the false discovery rate at *q* = 0.05.

Specification of the multivariable multinomial logistic regression model. The dependent variable was AD severity defined by CDR, analyzed as a nominal three-level outcome (early-stage, moderate, and severe) with early-stage AD as the reference category; results are therefore reported as two sets of coefficients (moderate vs. early-stage and severe vs. early-stage). Seven candidate predictors were considered: MMSE, ADL, estimated symptom duration, plasma GFAP, and the three TCM syndrome element scores (Kidney deficiency, phlegm stagnation, and heat toxin). MoCA was excluded due to collinearity with MMSE. A multinomial rather than an ordinal model was used because the proportional odds assumption was violated (omnibus LR test: χ^2^ = 19.61, df = 7, *p* = 0.0065), supporting use of a multinomial model. The overall model test was significant (χ^2^ = 301.776, df = 14, *p* < 0.001). Model fit statistics: −2 log likelihood = 170.23, AIC = 202.23, BIC = 257.65, McFadden’s pseudo R^2^ = 0.639.

Model adequacy and diagnostics. The linearity assumption for the seven continuous predictors was examined using a modified (shifted) Box–Tidwell procedure, in which each predictor X is supplemented by the product term X × ln(X+1). Several predictors in this dataset contained zero values—most notably MMSE, which can legitimately take a score of 0. Although the conventional Box–Tidwell term X × ln(X) is undefined at X = 0, its limit as X→0^+^ is 0. To retain all observations and evaluate linearity across the full range of each predictor without ad hoc exclusion or imputation, we adopted the shifted transformation X × ln(X+1), which is well-defined at zero (with value 0) and preserves the exploratory purpose of the procedure. Each term was evaluated by a likelihood ratio test across both logits (df = 2 per test), against a Bonferroni-corrected threshold (α = 0.05/7 = 0.0071). Six predictors showed no evidence of departure from linearity (all *p* > 0.0071). ADL showed a possible departure (*p* = 0.00024) and was therefore re-examined using a three-knot restricted cubic spline (knots at the 10th, 50th, and 90th percentiles); the non-linear component, tested jointly across both logits, was not statistically significant (χ^2^ = 0.458, df = 2, *p* = 0.795), indicating that a linear term was sufficient. All continuous predictors were therefore entered in linear form.

Because a global CDR score of 0.5 denotes very mild or questionable dementia and is the stage at which staging is least certain, all of the above analyses were repeated as a sensitivity analysis after excluding participants with CDR = 0.5, including group comparisons, correlation analyses and the multivariable multinomial logistic regression, using the same model specification and diagnostic procedures (Supplementary Tables 6-1–6-4). In the sensitivity regression, the multinomial logistic regression model was fitted with AD stage (early-stage vs. moderate vs. severe; early-stage as reference) as the outcome and MMSE, ADL, estimated symptom duration, GFAP, kidney essence deficiency, phlegm and stasis, and heat toxin as predictors; MoCA was excluded due to collinearity with MMSE. Given violation of the proportional odds assumption (omnibus LR test: χ^2^ = 15.79, df = 7, *p* = 0.027), a multinomial rather than ordinal model was adopted. The overall model test was significant (χ^2^ = 237.775, df = 14, *p* < 0.001). Model fit statistics: AIC = 192.76; McFadden’s pseudo R^2^ = 0.597.

## 3 Results

A total of 236 participants [among 236 AD patients, 38 (16.1%) had early-onset AD (onset < 65 years)] were included in this study. based on western medical diagnostic criteria, 127 individuals (53.81%) were classified as having early-stage AD, 66 (27.97%) as moderate AD, and 43 (18.22%) as severe AD. According to TCM syndrome differentiation, 87 participants (36.86%) were identified with kidney essence deficiency syndrome, 93 (39.41%) with Phlegm-stasis obstructing the orifices syndrome, and 56 (23.73%) with Heat-toxin obstructing the collaterals syndrome (Figure 3).

**FIGURE 3:**
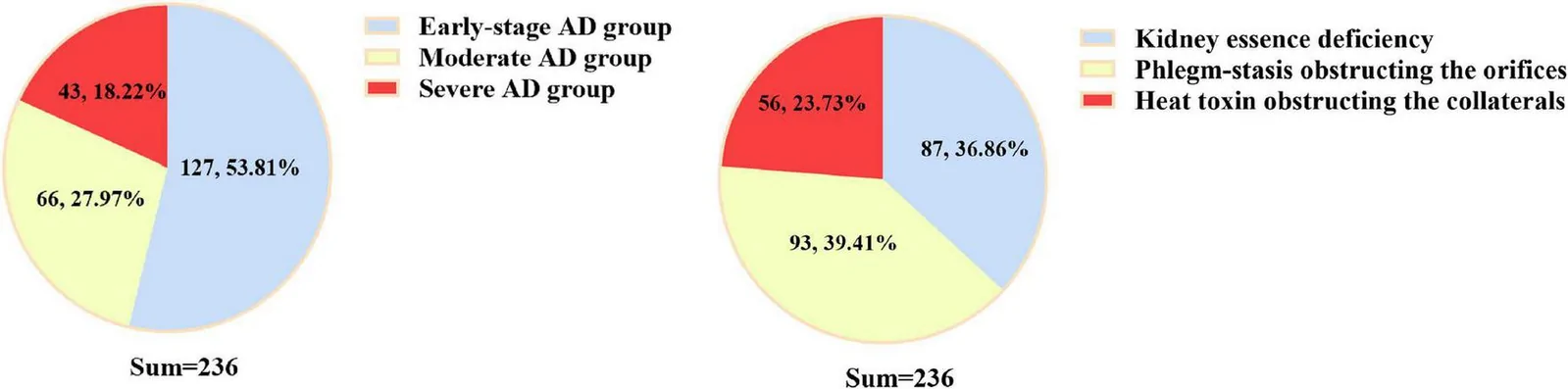
Composition of Western Medicine and Traditional Chinese Medicine (TCM) groupings among enrolled Alzheimer’s disease (AD) cases.

### 3.1 Analysis of clinical data in patients with early-stage, moderate, and severe AD

#### 3.1.1 Comparison of general information among patients in the early-stage, moderate, and severe AD groups

Statistically significant differences were observed among the three patient groups in terms of MMSE, MoCA, ADL, and estimated symptom duration (*p* < 0.05, q < 0.05). In contrast, no statistically significant differences were found regarding age, years of education, depression, gender, smoking, alcohol consumption, hypertension, heart disease, hyperlipidemia, and family history of dementia (*p* > 0.05) (Supplementary Table 1) (*n* = 236).

After excluding CDR = 0.5 participants, MMSE, MoCA, ADL and estimated symptom duration differed significantly across the early-stage (*n* = 77), moderate (*n* = 66) and severe (*n* = 43) AD groups (MMSE: *p* < 0.001, q < 0.001; MoCA: *p* < 0.001, q < 0.001; ADL: *p* < 0.001, q < 0.001; estimated symptom duration: *p* < 0.001, q < 0.001) (Supplementary Table 6-2) (*n* = 186).

#### 3.1.2 Comparative analysis of peripheral blood biomarkers across early-stage, moderate, and severe AD groups

In this cross-sectional sample, unadjusted GFAP concentrations appeared to differ across severity groups, with higher values observed in the severe group (*p* = 0.025); however, this difference did not survive correction for multiple comparisons (q = 0.060). No statistically significant differences were observed in NfL, p-tau181, p-tau217, and Aβ42/40 (*p* > 0.05) (Table 1) (*n* = 236).

**TABLE 1:** Comparison of peripheral blood biomarkers among patients with early-stage, moderate, and severe Alzheimer’s disease (AD).

Characteristic	Early-stage (*n* = 127)	Moderate (*n* = 66)	Severe (*n* = 43)	H	*P*	*q*
GFAP	11.48 (7.18, 18.72)	13.0 (9.30, 21.88)	17.4 (9.71, 29.24)	7.39	0.025	0.060
Nfl	8.86 (3.56, 15.80)	11.18 (5.38, 17.57)	13.55 (6.61, 23.93)	5.64	0.059	0.119
p-tau181	4.02 (2.33, 6.07)	4.24 (2.51, 6.38)	4.03 (1.94, 7.38)	0.81	0.666	0.667
p-tau217	6.00 (2.11, 9.06)	6.90 (3.01, 9.31)	6.88 (3.06, 11.28)	1.80	0.407	0.488
Aβ42/40	0.32 (0.10, 0.66)	0.32 (0.15, 0.89)	0.35 (0.15, 0.75)	0.81	0.667	0.667

After excluding CDR = 0.5 participants, the plasma biomarkers P-tau181 (*p* = 0.995, q = 0.995), P-tau217 (*p* = 0.833, q = 0.980), Aβ42/40 (*p* = 0.543, q = 0.836), GFAP (*p* = 0.015, q = 0.061) and NFL (*p* = 0.037, q = 0.106), showed no statistically significant differences after FDR correction (Supplementary Table 6-2) (*n* = 186).

#### 3.1.3 Correlation analysis between dementia severity levels and differential indicators in AD

This study aims to perform a correlation analysis of significant variables derived from general information and peripheral blood biomarkers in relation to early-stage, moderate, and severe AD. The results indicate strong negative correlations between dementia severity and both MMSE (*r* = −0.779, *p* < 0.001) and MoCA scores (*r* = −0.785, *p* < 0.001), indicating worse cognitive function with advancing disease. A strong positive correlation was observed with ADL scores (*r* = 0.742, *p* < 0.001), reflecting greater functional impairment in severe stages. Weak positive correlations were found with estimated symptom duration (*r* = 0.384, *p* < 0.001) (Table 2) (*n* = 236).

**TABLE 2:** Correlation analysis of different degrees of dementia and various differential indicators in Alzheimer’s disease (AD).

Variable	*r*	95% CI	*P*
MMSE	−0.779	−0.825∼−0.723	<0.001
MoCA	−0.785	−0.830∼−0.730	<0.001
ADL	0.742	0.679∼0.794	<0.001
Estimated symptom duration	0.384	0.269∼0.488	<0.001
GFAP	0.175	0.048∼0.296	0.007

—r| ≥ 0.6 indicates a strong correlation, 0.6 > —r| ≥ 0.4 signifies a moderate correlation, and —r| < 0.4 denotes a weak correlation.

After excluding participants with CDR = 0.5, the spearman correlation analysis indicated MMSE (*r* = −0.766, *p* < 0.001) and MoCA (*r* = −0.771, *p* < 0.001) showed strong negative correlations with dementia severity, whereas ADL (*r* = 0.716, *p* < 0.001) showed a strong positive correlation and estimated symptom duration (*r* = 0.362, *p* < 0.001) and GFAP (*r* = 0.212, *p* = 0.004) showed weak positive correlations with dementia severity (Supplementary Table 6-3) (*n* = 186).

### 3.2 Distribution patterns of TCM syndromes across early-stage, moderate, and severe AD groups

#### 3.2.1 Comparison of the distribution frequency and characteristics of TCM syndromes among the three patient groups

Analysis of TCM syndrome patterns in 236 AD patients revealed distinct distribution trends across severity stages. In early-stage AD, syndrome frequency decreased as follows: Kidney essence deficiency > Phlegm-stasis obstructing the orifices > Heat-toxin obstructing the collaterals. Moderate AD showed: Phlegm-stasis obstructing the orifices > kidney essence deficiency > Heat-toxin obstructing the collaterals, while severe AD exhibited: Heat-toxin obstructing the collaterals > Phlegm-stasis obstructing the orifices > kidney essence deficiency (Figure 4).

**FIGURE 4:**
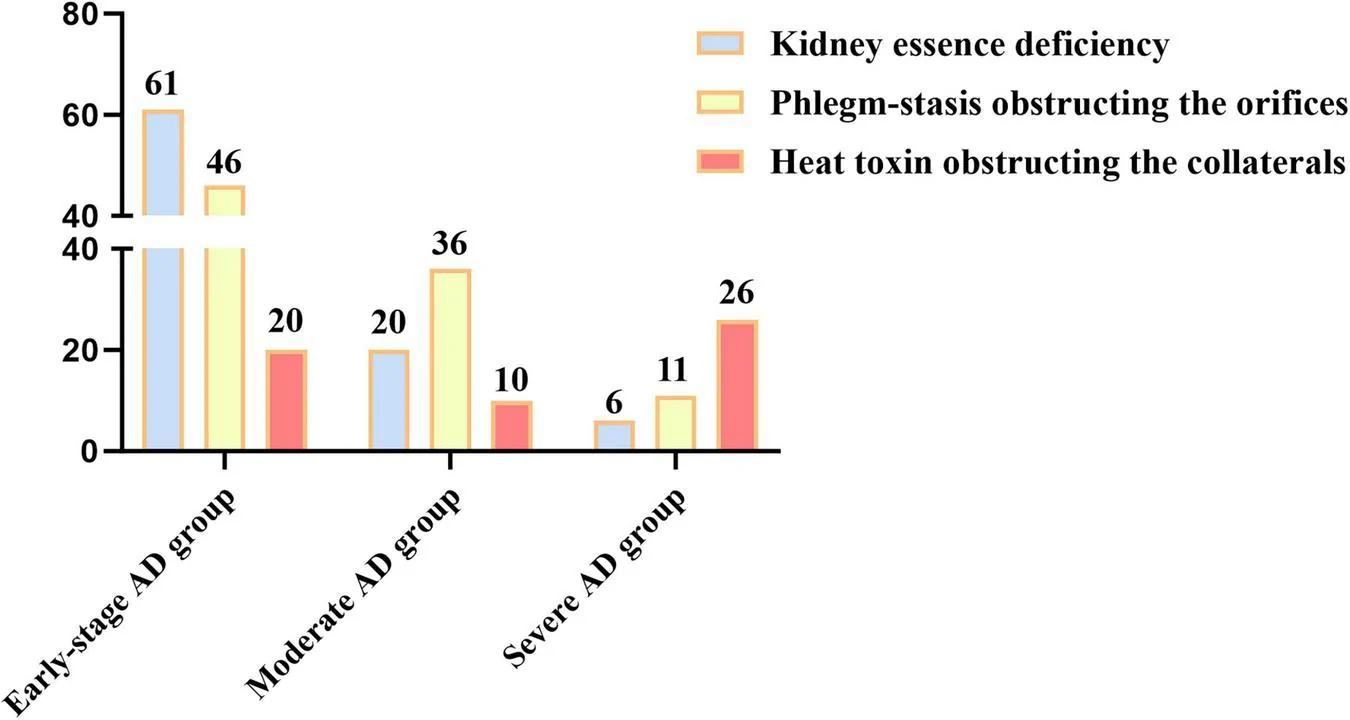
Distribution of Alzheimer’s disease (AD) patients by western medicine severity and Traditional Chinese Medicine (TCM) syndrome type.

Chi-square analysis demonstrated significant differences in syndrome distribution across severity groups. In the early-stage group, kidney essence deficiency was the most frequent pattern (48.0%) but did not differ significantly from Phlegm-stasis obstructing the orifices (36.2%); both were significantly more frequent than Heat-toxin obstructing the collaterals (15.7%). In the moderate group, Phlegm-stasis obstructing the orifices predominated (54.5%), being significantly more frequent than Heat-toxin obstructing the collaterals (15.2%); kidney essence deficiency (30.3%) was intermediate and did not differ significantly from either of the other two patterns. In the severe group, Heat-toxin obstructing the collaterals predominated (60.5%), exceeding both kidney essence deficiency (14.0%) and Phlegm-stasis obstructing the orifices (25.6%), which did not differ significantly from each other (Supplementary Table 2).

#### 3.2.2 Comparison of TCM syndrome scores among the three patient groups

When comparing the early-stage TCM syndrome scores of AD with those of moderate AD, the scores for kidney essence deficiency exhibited no significant difference. In contrast, the scores for phlegm and stasis obstructing the orifices, as well as Heat toxin obstructing the channels, demonstrated significant differences (*p* < 0.01). Compared to the early-stage and moderate TCM syndrome scores in the AD groups, the severe AD group exhibited significant differences in scores for kidney essence deficiency, phlegm and stasis obstructing the orifices, and Heat toxin obstructing the channels (*p* < 0.01) (Table 3) (*n* = 236).

**TABLE 3:** Comparison of Traditional Chinese Medicine (TCM) Syndrome scores among patients with early-stage, moderate, and severe Alzheimer’s disease (AD).

Group	Case count	TCM syndrome type		
		Kidney essence deficiency	Phlegm and stasis obstructing the orifices	Heat-toxin obstructing the channels
Early-stage	127	11.0 (8.0, 15.0)	11.0 (7.0, 14.0)	5.0 (3.0, 8.0)
Moderate	66	11.0 (7.0, 15.0)	14.0 (9.0, 16.0)	7.0 (5.0, 11.75)
Severe	43	5.0 (5.0, 11.5)	8.0 (5.0, 13.5)	14.0 (10.5, 17.0)
χ^2^	–	23.31	21.44	67.48
*p*	–	<0.001	<0.001	<0.001

### 3.3 Analysis of clinical data in patients with different TCM syndrome types

#### 3.3.1 Comparative analysis of general characteristics among patients with kidney essence deficiency, Phlegm-stasis obstructing the orifices, and Heat-toxin obstructing the collaterals syndromes

The differences in Estimated symptom duration, MMSE, MoCA, ADL, and CDR among the three groups of patients diagnosed with different TCM syndromes were statistically significant (*p* < 0.05). In contrast, there were no statistically significant differences in age, years of education, depression, heart disease, family history of dementia, gender, smoking, alcohol consumption, hypertension, diabetes, and hyperlipidemia (*p* > 0.05) (Supplementary Table 3) (*n* = 236).

After excluding participants with CDR = 0.5, the three TCM syndrome groups (kidney essence deficiency, *n* = 65; Phlegm-stasis obstructing the orifices, *n* = 69; heat toxin obstructing the channels, *n* = 52) were comparable at baseline, and no variable showed a statistically significant difference after FDR correction (all q > 0.05 before correction, MMSE *p* = 0.008, q = 0.073; MoCA *p* = 0.011, q = 0.073; ADL *p* = 0.008, q = 0.073; estimated symptom duration *p* = 0.020, q = 0.100; diabetes *p* = 0.046, q = 0.149; hyperlipidemia *p* = 0.042, q = 0.149; all other variables *p* > 0.05) (Supplementary Table 6-1) (*n* = 186).

#### 3.3.2 Comparison of peripheral blood biomarkers among patients with kidney essence deficiency, Phlegm-stasis obstruction, and Heat-toxin obstruction syndromes

Statistically significant differences in peripheral blood p-tau217 levels were observed among the three groups of patients diagnosed with different TCM syndromes (*p* < 0.05). However, this difference did not survive correction for multiple comparisons (q > 0.05). No statistically significant differences were observed in GFAP, NfL, p-tau181, and Aβ42/40 levels (*p* > 0.05) (Supplementary Table 4) (*n* = 236).

After excluding participants with CDR = 0.5, no statistically significant differences were observed across the three TCM syndrome groups in any of the plasma biomarkers, including GFAP (*p* = 0.123, q = 0.273), NFL (*p* = 0.247, q = 0.412), P-tau181 (*p* = 0.662, q = 0.858), P-tau217 (*p* = 0.238, q = 0.412) and Aβ42/40 (*p* = 0.968, q = 0.968) (Supplementary Table 6-1) (*n* = 186).

#### 3.3.3 Correlation analysis between different TCM syndromes and various differential indicators

The significant variables identified through differential analysis were correlated with various TCM syndromes. The results indicated that kidney deficiency and Phlegm-stasis were not significantly correlated with MMSE, MoCA, ADL, estimated symptom duration, or CDR. in contrast, Heat-toxin showed a significant negative correlation with MMSE and MoCA (*r* = −0.376 and −0.369, respectively, *p* < 0.01). Furthermore, ADL and CDR exhibited a significant positive correlation with Heat-toxin (*r* = 0.271 and 0.346, respectively, *p* < 0.05) (Table 4) (*n* = 236).

**TABLE 4:** Correlation analysis between different Traditional Chinese Medicine (TCM) syndromes and various differential indicators.

Variable	Kidney deficiency (*n* = 87)			Phlegm-stasis (*n* = 93)			Heat-toxin (*n* = 56)		
	*r*	95% CI	*P*	*r*	95% CI	*P*	*r*	95% C*I*	*P*
MMSE	−0.075	(−0.281, 0.138)	0.488	−0.049	(−0.25, 0.156)	0.643	−0.376	(−0.581, −0.126)	0.004
Moca	−0.037	(−0.246, 0.175)	0.731	−0.073	(−0.273, 0.133)	0.491	−0.369	(-0.576, -0.117)	0.006
ADL	0.000	(−0.211, 0.211)	1.000	−0.005	(−0.208, 0.199)	0.961	0.271	(0.009, 0.498)	0.043
Estimated symptom duration	0.032	(−0.18, 0.241)	0.772	−0.019	(−0.222, 0.185)	0.858	−0.180	(−0.423, 0.087)	0.190
CDR	0.107	(−0.106, 0.311)	0.325	0.134	(−0.072, 0.329)	0.201	0.346	(0.089, 0.560)	0.010

A correlation coefficient of —r| ≥ 0.6 signifies a strong correlation; 0.6 > —r| ≥ 0.4 indicates a moderate correlation; and —r| < 0.4 reflects a weak correlation.

### 3.4 Logistic regression analysis of factors associated with disease severity in patients with early-stage, moderate, and severe AD

Multivariable multinomial logistic regression was performed with early-stage AD as the reference category. MMSE scores were inversely associated with AD severity: higher MMSE scores corresponded to lower odds of being classified into a more advanced stage (moderate vs. early: OR = 0.844, 95% CI 0.774–0.921, p < 0.001; severe vs. early: OR = 0.485, 95% CI 0.351–0.671, p < 0.001), and this association was stronger in the severe group. In contrast, ADL scores were positively associated with AD severity (moderate vs. early: OR = 1.128, 95% CI 1.065–1.196, p < 0.001; severe vs. early: OR = 1.280, 95% CI 1.150–1.425, p < 0.001) (Table 5) ( n = 236).

**TABLE 5:** Logistic regression analysis of factors associated with Alzheimer’s disease (AD) severity.

Characteristic	Variable	β	S.E.	Wald χ^2^	*P*	Exp(B)	95% CI for Exp(B)
Moderate AD vs. early-stage AD	MMSE	−0.169	0.044	14.509	<0.001	0.844	0.774∼0.921
	ADL	0.121	0.030	16.547	<0.001	1.128	1.065∼1.196
	Estimated symptom duration	0.016	0.055	0.090	0.765	1.017	0.913∼1.133
	GFAP	0.010	0.010	0.938	0.333	1.010	0.990 ∼ 1.031
	Kidney deficiency	0.017	0.045	0.138	0.710	1.017	0.931∼1.110
	Phlegm stagnation	0.180	0.053	11.531	0.001	1.198	1.079∼1.329
	Heat-toxin	0.163	0.060	7.308	0.007	1.177	1.046∼1.324
Severe AD vs. early-stage AD	MMSE	−0.723	0.166	19.059	<0.001	0.485	0.351∼0.671
	ADL	0.247	0.055	20.451	<0.001	1.280	1.150∼1.425
	Estimated symptom duration	−0.029	0.070	0.170	0.680	0.972	0.848∼1.114
	GFAP	0.027	0.022	1.508	0.220	1.027	0.984∼1.073
	Kidney deficiency	−0.119	0.113	1.110	0.292	0.888	0.711∼1.108
	Phlegm stagnation	0.182	0.102	3.211	0.073	1.200	0.983∼1.464
	Heat-toxin	0.404	0.111	13.293	<0.001	1.498	1.205∼1.861

A multinomial logistic regression model was fitted with AD stage (early-stage as reference) as the outcome and MMSE, activities of daily living (ADL), estimated symptom duration, glial fibrillary acidic protein (GFAP), kidney essence deficiency, phlegm-stasis, and heat toxin as predictors; MoCA was excluded due to collinearity with MMSE. The proportional odds assumption was violated (omnibus LR test: χ^2^ = 19.61, df = 7, *p* = 0.0065), supporting use of a multinomial model. The overall model test was significant (χ^2^ = 301.776, df = 14, *p* < 0.001). Model fit statistics: −2 log likelihood = 170.23, AIC = 202.23, BIC = 257.65, McFadden’s pseudo R^2^ = 0.639.

Among the TCM syndrome elements, phlegm stagnation was significantly associated with moderate AD (OR = 1.198, 95% CI 1.079–1.329, p = 0.001) and showed a consistent but non-significant trend toward severe AD (OR = 1.200, 95% CI 0.983–1.464, p = 0.073). Heat toxin was significantly associated with both moderate and severe AD, with a stronger effect observed in the severe group (moderate: OR = 1.177, 95% CI 1.046–1.324, p = 0.007; severe: OR = 1.498, 95% CI 1.205–1.861, p < 0.001). Kidney deficiency was not significantly associated with either comparison ( p = 0.710 and p = 0.292, respectively). Neither serum GFAP level nor estimated symptom duration showed a significant association with AD severity in either model (all p > 0.05) (Table 5) ( n = 236).

After excluding participants with CDR = 0.5, Multivariable multinomial logistic regression was performed with early-stage AD as the reference category (*n* = 186). MMSE scores were inversely associated with AD severity: higher MMSE scores corresponded to lower odds of being classified into a more advanced stage (moderate vs. early: OR = 0.86, 95% CI 0.79–0.94, *p* < 0.001; severe vs. early: OR = 0.50, 95% CI 0.37–0.69, *p* < 0.001), and this association was stronger in the severe group. In contrast, ADL scores were positively associated with AD severity (moderate vs. early: OR = 1.12, 95% CI 1.05–.12, 9*p* < 0.001; severe vs. early: OR = 1.27, 95% CI 1.14 early:*p* < 0.001) (Supplementary Table 6-4). Among the TCM syndrome elements, phlegm stagnation was significantly associated with both moderate and severe AD, with comparable effect sizes across the two comparisons (moderate: OR = 1.26, 95% CI 1.12M syndr*p* < 0.001; severe: OR = 1.25, 95% CI 1.0225, 95%*p* = 0.032). Heat toxin was also significantly associated with both moderate and severe AD, with a stronger effect observed in the severe group (moderate: OR = 1.20, 95% CI 1.06n1.35, *p* = 0.004; severe: OR = 1.50, 95% CI 1.225% ere:*p* < 0.001). Kidney deficiency was not significantly associated with either comparison (*p* = 0.522 and *p* = 0.287, respectively). Neither serum GFAP level nor estimated symptom duration showed a significant association with AD severity in either comparison (all *p* > 0.05) (Supplementary Table 6-4) (*n* = 186).

### 3.5 ROC curve analysis for exploratory discriminatory performance

Indicators associated with both CDR-defined severity and TCM syndrome patterns in the preceding correlation analyses (MMSE, MoCA, ADL, estimated symptom duration, and CDR) were entered into an exploratory ROC analysis. This analysis aimed to characterize the discriminatory performance of these indicators for TCM syndrome patterns in an exploratory manner, rather than to establish diagnostic accuracy.

#### 3.5.1 ROC curve analysis of the discriminatory performance of related indicators for different TCM syndrome types

The exploratory evaluation of clinical indicators for discriminating TCM syndrome patterns revealed distinct discriminatory profiles across assessment tools. The MMSE showed a modest AUC for the Heat-toxin pattern (AUC = 0.683, 95% CI: 0.596–0.770), whereas the AUCs for Kidney deficiency (AUC = 0.595, 95% CI: 0.522–0.668) and Phlegm-stasis (AUC = 0.546, 95% CI: 0.472–0.621) were close to chance. The ADL scale yielded a slightly higher AUC for the Heat-toxin pattern (AUC = 0.690, 95% CI: 0.608–0.772), but remained uninformative for Kidney deficiency (AUC = 0.590, 95% CI: 0.518–0.666) and Phlegm-stasis (AUC = 0.554, 95% CI: 0.480–0.629). Estimated symptom duration yielded a marginal AUC for the Heat-toxin pattern (AUC = 0.647, 95% CI: 0.561–0.734) and AUCs close to chance for Kidney deficiency (AUC = 0.530, 95% CI: 0.456–0.605) and Phlegm-stasis (AUC = 0.581, 95% CI: 0.507–0.656). The CDR scale showed the highest single-indicator AUC for the Heat-toxin pattern (AUC = 0.709, 95% CI: 0.630–0.787), numerically higher than MMSE and ADL, while remaining uninformative for Kidney deficiency (AUC = 0.633, 95% CI: 0.564–0.701) and Phlegm-stasis (AUC = 0.471, 95% CI: 0.398–0.544) (Figure 5 and Supplementary Table 5). These estimates were derived from a single development sample without external validation and should be interpreted as exploratory.

**FIGURE 5:**
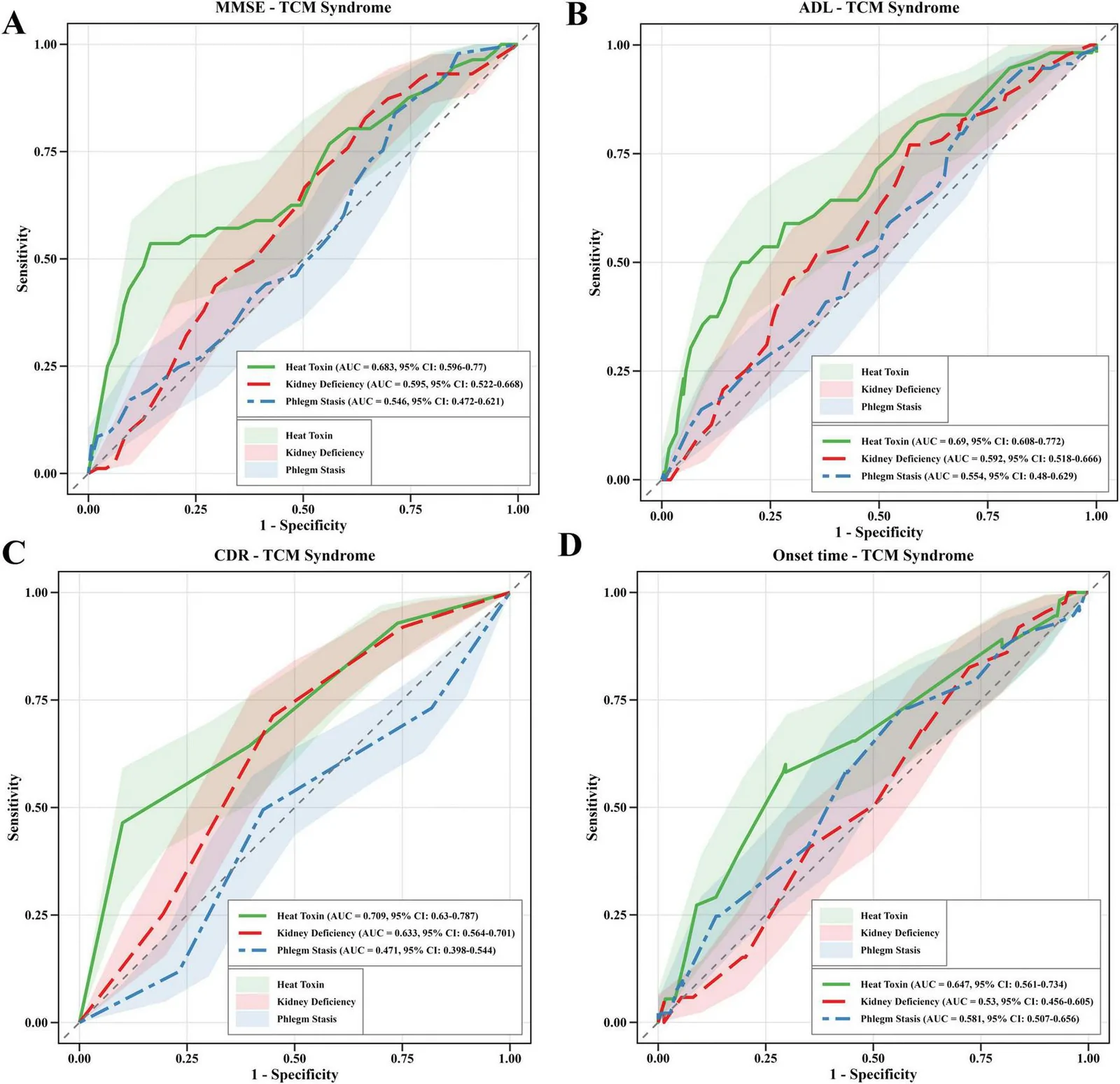
Exploratory ROC curve analysis of the discriminatory performance of relevant indicators for Traditional Chinese Medicine (TCM) syndrome patterns. **(A)** ROC curve analysis of MMSE indicators. **(B)** ROC curve analysis of ADL indicators. **(C)** ROC curve analysis of CDR indicators. **(D)** ROC curve analysis of estimated symptom duration (onset time) indicators.

## 4 Discussion

Alzheimer’s disease remains a major challenge in both diagnostic and therapeutic research (Hardy, 2025; Scheltens et al., 2021). The prevailing international framework, anchored in the NIA-AA criteria, has largely focused on cerebral Aβ, tau pathology, and neurodegeneration biomarkers (Jack et al., 2018). Recent advances in plasma biomarker detection—particularly Simoa-based assays for p-tau217 and GFAP—have opened new avenues for early diagnosis and monitoring (Pais et al., 2023). Yet these biomarkers have not fully elucidated the clinical heterogeneity of AD, and Western biomedical indicators alone appear insufficient to capture the complexity of the disease course. In China, growing attention has turned to integrative approaches combining Western and TCM perspectives. TCM conceptualizes AD under the rubric of “dementia” and posits a framework of “Kidney deficiency as the root, Phlegm-stasis as the manifestation, and toxin injury to the brain collaterals as the pathological consequence” (Chen et al., 2018; Hardy, 2025). Drawing on this theoretical background, the present cross-sectional study integrated TCM syndrome differentiation (Kidney deficiency, Phlegm-stasis, and Heat-toxin) with conventional cognitive assessments (MMSE, MoCA, ADL) and CDR staging to explore the concurrent associations between disease severity and TCM syndrome patterns, and to generate hypotheses for future research.

In our analysis of 236 AD patients, we examined factors associated with disease severity. Significant differences emerged across severity groups in MMSE, MoCA, ADL, and Estimated symptom duration, whereas age, education, sex, and medical history showed no significant associations. MMSE and MoCA scores demonstrated strong inverse correlations with severity, while ADL showed a strong positive correlation, underscoring functional impairment as a key feature of clinical deterioration. Estimated symptom duration exhibited a weak positive correlation, which may reflect longer illness duration co-occurring with greater functional decline in this cross-sectional sample. Among plasma biomarkers, only GFAP showed a weak positive correlation with severity, consistent with its recognized role as a marker of astroglial activation or injury (Kim et al., 2023). No significant associations were observed for NfL, p-tau181, p-tau217, or the Aβ42/40 ratio. Although NfL and p-tau181 are well-established diagnostic markers, their staging value remains debated; our negative findings are consistent with some reports (Pilotto et al., 2025). In the multivariable multinomial logistic regression (reference: early-stage AD), lower MMSE and higher ADL were independently associated with greater severity, with stronger effects for severe than moderate AD (MMSE: OR = 0.485 vs. 0.844; ADL: OR = 1.280 vs. 1.128; all *p* < 0.001). Among TCM syndrome elements, heat toxin was significant in both groups (severe: OR = 1.498, *p* < 0.001; moderate: OR = 1.177, *p* = 0.007) and phlegm stagnation only in the moderate group (OR = 1.198, *p* = 0.001), whereas Kidney deficiency, GFAP and symptom duration were all non-significant (*p* > 0.05).

Analysis of TCM syndrome patterns revealed that the distribution and scores of Kidney essence deficiency, Phlegm-stasis, and Heat-toxin differed significantly across severity stages, broadly consistent with the TCM framework of “Kidney deficiency as the root, Phlegm-stasis as the manifestation, and toxin injury as the pathological consequence” (Dong et al., 2025). Kidney essence deficiency predominated in early-stage AD (48%), aligning with the principle that “the kidney governs marrow and connects to the brain” (Zhang et al., 2024). In moderate AD, Phlegm-stasis increased substantially, reflecting gradual accumulation of pathological products. In severe AD, Heat-toxin emerged as the dominant syndrome, corresponding to clinical presentations such as agitation and mania, consistent with the theorized pattern of “prolonged illness entering the collaterals and transforming into toxin” (Dong et al., 2025). Chi-square tests confirmed that the distribution differences for Phlegm-stasis and Heat-toxin were stage-specific. Syndrome scores for Heat-toxin increased markedly with severity, while Phlegm-stasis scores peaked in the moderate group (median 14.0) and then declined in the severe group (median 8.0), although the overall difference across the three severity stages remained significant (Kruskal–Wallis *p* < 0.001, Table 3). Kidney deficiency scores declined significantly only in the severe stage. These findings are consistent with the hypothesis that Kidney deficiency represents the underlying predisposition, while the interplay of phlegm, stasis, and toxin is associated with more advanced clinical stages. Notably, Kidney deficiency scores did not change significantly from early-stage to moderate stages, possibly because prominent Phlegm-stasis manifestations mask the underlying deficiency. An alternative interpretation is that syndrome scoring partly relies on patient symptom report and clinical examinability, both of which diminish with advancing dementia severity; thus, differential measurement across severity strata may also contribute to the observed decline in Kidney deficiency scores. Modern pharmacological studies have shown that kidney-tonifying herbs modulate Aβ metabolism (Qi et al., 2012) and phlegm-resolving, stasis-invigorating formulas improve cerebral microcirculation, providing biological plausibility for syndrome-based treatment. The observed syndrome distribution pattern—from Kidney deficiency to Phlegm-stasis to Heat-toxin—is suggestive of a pathological cascade from deficiency to excess to toxin, though the cross-sectional design of the present study precludes conclusions regarding temporal sequencing. Future longitudinal studies are required to test this hypothesized sequence.

Comparison of the three TCM syndrome groups revealed significant differences in MMSE, MoCA, ADL, CDR, and Estimated symptom duration, but not in age or comorbidities. Kidney essence deficiency patients showed relatively preserved cognition and function, consistent with early AD (Zhang et al., 2024). Phlegm-stasis patients showed intermediate impairment, and Heat-toxin patients showed the most severe cognitive and functional deficits, consistent with the “toxin injury to brain collaterals” framework (Dong et al., 2025). In the correlation analysis, neither Kidney deficiency nor Phlegm-stasis was significantly associated with MMSE, MoCA, ADL, estimated symptom duration, or CDR. By contrast, Heat-toxin correlated negatively with MMSE (*r* = −0.376) and MoCA (*r* = −0.369) (both *p* < 0.01) and positively with ADL (*r* = 0.271) and CDR (*r* = 0.346) (both *p* < 0.05). Furthermore, ADL and CDR exhibited a significant positive correlation with Heat-toxin (*r* = 0.271 and 0.346, respectively, *p* < 0.05). Among plasma biomarkers, unadjusted p-tau217 levels appeared higher in the Heat-toxin group, but this difference did not survive FDR correction (corrected q > 0.05; Supplementary Table 4). GFAP, NfL, and the Aβ42/40 ratio also showed no significant differences across TCM syndrome groups. The nominal elevation of p-tau217 in the Heat-toxin group, though not robust to correction, is hypothesis-generating and warrants replication in larger cohorts. This finding parallels our earlier observation that p-tau217 did not differ across CDR stages. The consistency of null results across both classification systems suggests that p-tau217 may have limited discriminatory value within the moderate-to-severe AD spectrum, consistent with the recognized plateau of phosphorylated tau in advanced disease (Kim et al., 2023). Future studies with larger samples and pre-clinical or early-stage AD participants may be better powered to detect whether TCM syndrome patterns capture tau-related heterogeneity beyond conventional staging.

To assess whether these findings depended on the inclusion of very mild cases, we repeated the principal analyses after excluding participants with CDR 0.5on the inclusion of very mild cases, we ture tau-clinical contrast with advanced disease is weakest (*n* = 186; early-stage 77, moderate 66, severe 43; Supplementary Tables 6-1–6-4). Most results were essentially unchanged: MMSE, MoCA and ADL remained strongly correlated with severity (—r| = 0.716ADL remained*p* < 0.001); the regression estimates for MMSE (moderate: OR = 0.86; severe: OR = 0.50) and ADL (moderate: OR = 1.12; severe: OR = 1.27) were virtually identical to those in the full sample; Kidney deficiency remained unassociated with severity; Heat-toxin remained significant for both comparisons, again with a stronger effect in severe than in moderate AD (OR = 1.20, *p* = 0.004; OR = 1.50, *p* < 0.001); and plasma biomarkers again showed no significant differences after FDR correction, with GFAP retaining only a weak correlation with severity (*r* = 0.212, *p* = 0.004). Two results did change. First, Phlegm-stasis became significantly associated with both moderate and severe AD (OR = 1.26, *p* < 0.001; OR = 1.25, *p* = 0.032), indicating that its restriction to the moderate stage in the primary analysis was not robust and was likely diluted by the CDR 0.5 participants retained in the early-stage reference group. Second, the differences in MMSE, MoCA, ADL and estimated symptom duration across the three TCM syndrome groups were attenuated and no longer survived FDR correction (all q > 0.05), suggesting that they were partly driven by the mildest participants. Overall, therefore, the severity-related cognitive, functional and Heat-toxin associations appear robust to the inclusion of very mild cases, whereas the stage-specificity of phlegm-stasis and the between-syndrome differences in clinical measures should be interpreted cautiously. Because these analyses included only participants with CDR ≥ 1.0, the findings apply to established dementia stages and should not be extended to the prodromal or very mild end of the AD spectrum.

In our analysis of 236 AD patients, we selected indicators associated with TCM syndrome types in the correlation analysis (MMSE, MoCA, ADL, disease Estimated symptom duration, and CDR) for ROC curve analysis to evaluate their exploratory discriminatory performance within this development sample. Notably, MMSE and MoCA showed high collinearity in preliminary analysis, thus MoCA was excluded from the subsequent modeling to avoid multicollinearity bias. The results indicate that single indicators showed only modest AUCs for the “Heat-Toxin” syndrome type, with ADL showing the best conventional scale performance (AUC = 0.690, 95% CI: 0.608–0.772) and CDR demonstrating the highest overall discriminatory power (AUC = 0.709, 95% CI: 0.630 (AUCn). In contrast, all single indicators yielded AUCs < 0.65 and were therefore uninformative for “Kidney Deficiency” and “Phlegm-Stasis” syndrome types. The numerically highest AUC observed for CDR is hypothesis-generating at most. However, these exploratory findings are derived from a single-center cohort without external validation; thus, subsequent multi-center studies with independent validation cohorts are warranted to confirm whether these observations are reproducible.

In summary, the present study observed that TCM syndrome distributions and scores differed across concurrent CDR-defined severity groups in this single-center sample. Heat-toxin scores were associated with greater concurrent cognitive and functional impairment, whereas plasma biomarkers showed limited and inconsistent associations. These findings do not establish temporal syndrome transitions, progression risk or validated diagnostic utility outside this development sample but provide hypothesis-generating evidence for future prospective investigations.

## Data Availability

The datasets presented in this study can be found in online repositories. The names of the repository/repositories and accession number(s) can be found in the article/Supplementary material.
