# Supplementary material for: Cross-sectional associations of Kidney deficiency, Phlegm-stasis, and Heat-toxin syndrome patterns with disease severity in Alzheimer’s disease: an exploratory study
Source: Front Aging Neurosci. 2026 Oct 16;18:1763273. doi: 10.3389/fnagi.2026.1763273 (PMC13719365; doi:10.3389/fnagi.2026.1763273)
Supplement: Supplementary file 1 [file Data_Sheet_1.docx]

**TABLE S1** Comparison of general information among patients with early-stage, moderate, and severe AD

| Characteristic | Early-stage (n=127) | Moderate (n=66) | Severe (n=43) | χ²/H | *p* | *q* |
| --- | --- | --- | --- | --- | --- | --- |
| Age/years | 73.0 (69.0, 79.0) | 73.0 (65.0, 78.0) | 72.0 (66.0, 76.0) | 1.93 | 0.382 | 0.488 |
| Gender/case(%) |  |  |  | 0.02 | 0.992 | 0.992 |
| Male | 44 (34.65) | 22 (33.85) | 15 (34.88) |  |  |  |
| Female | 83 (65.35) | 43 (66.15) | 28 (65.12) |  |  |  |
| Years of education | 9.0 (9.0, 13.0) | 9.0 (6.0, 12.0) | 9.0 (7.5, 12.0) | 3.17 | 0.205 | 0.307 |
| Estimated symptom duration (years) | 2.0 (2.0, 4.0) | 4.0 (2.0, 5.0) | 6.0 (4.03, 8.0) | 36.59 | <0.001 | **<0.001** |
| Hypertension(%) | 56 (44.09) | 20 (30.30) | 16 (37.21) | 3.54 | 0.170 | 0.524 |
| Diabetes(%) | 24 (19.35) | 8 (12.12) | 14 (32.56) | 6.89 | 0.032 | **0.255** |
| Heart disease(%) | 17 (13.49) | 7 (10.77) | 4 (9.30) | 0.66 | 0.720 | 0.834 |
| Hyperlipidemia(%) | 27 (21.43) | 11 (16.67) | 9 (21.43) | 0.67 | 0.716 | 0.834 |
| GDS-30 | 4.0 (2.0, 8.25) | 3.0 (1.0, 10.5) | 2.0 (0.0, 7.0) | 3.47 | 0.176 | 0.302 |
| Smoking(%) | 23 (18.11) | 9 (13.64) | 3 (6.98) | 3.26 | 0.196 | 0.524 |
| Alcohol consumption(%) | 21 (16.67) | 10 (15.15) | 5 (11.63) | 0.63 | 0.730 | 0.834 |
| Family history of dementia | 30 (24.0) | 12 (18.46) | 10 (23.26) | 0.78 | 0.676 | 0.834 |
| MMSE | 19.0 (15.0, 23.0) | 9.0 (6.0, 12.0) | 1.0 (0.0, 3.5) | 143.51 | <0.001 | **<0.001** |
| MoCA | 13.0 (10.0, 16.0) | 4.0 (3.0, 7.0) | 1.0 (1.0, 1.75) | 143.81 | <0.001 | **<0.001** |
| ADL | 19.0 (16.0, 24.0) | 35.5 (30.0, 40.5) | 47.0 (43.0, 51.0) | 129.91 | <0.001 | **<0.001** |
