# Supplementary material for: Cross-sectional associations of Kidney deficiency, Phlegm-stasis, and Heat-toxin syndrome patterns with disease severity in Alzheimer’s disease: an exploratory study
Source: Front Aging Neurosci. 2026 Oct 16;18:1763273. doi: 10.3389/fnagi.2026.1763273 (PMC13719365; doi:10.3389/fnagi.2026.1763273)
Supplement: Supplementary file 2 [file Data_Sheet_2.docx]

**TABLE S2** Comparative analysis of TCM syndrome distribution patterns among patients with early-stage, moderate, and severe AD ^[13, 14]^

| Group | Case count | TCM syndrome type | | |
| --- | --- | --- | --- | --- |
|  |  | Kidney essence deficiency | Phlegm and stasis obstructing the orifices | Heat toxin obstructing the channels |
| Early-stage | 127 | 61^a^ (48.0%) / (70.1%) | 46^a^ (36.2%) / (49.5%) | 20^b^ (15.7%) / (35.7%)) |
| Moderate | 66 | 20^ab^ (30.3%) / (23.0%) | 36^a^ (54.5%) / (38.7%) | 10^b^ (15.2%) / (17.9%) |
| Severe | 43 | 6^a^ (14.0%) / (6.9%) | 11^a^ (25.6%) / (11.8%) | 26^b^ (60.5%) / (46.4%) |
| χ² | 47.35 | | | |
| *p* | <0.001 | | | |
| Note: Values are n (row %) / (column %). Superscript letters derive from pairwise comparisons of the counts, which account for the differing group sizes (127, 66, 43) and are therefore equivalent to comparisons of row percentages. Groups sharing the same superscript letter are not significantly different; different letters indicate a significant difference. | | | | |
