# Supplementary material for: Cross-sectional associations of Kidney deficiency, Phlegm-stasis, and Heat-toxin syndrome patterns with disease severity in Alzheimer’s disease: an exploratory study
Source: Front Aging Neurosci. 2026 Oct 16;18:1763273. doi: 10.3389/fnagi.2026.1763273 (PMC13719365; doi:10.3389/fnagi.2026.1763273)
Supplement: Supplementary file 3 [file Data_Sheet_3.docx]

**TABLE S3** Comparison of general data among patients with kidney essence deficiency, phlegm-stasis obstruction, and heat-toxin obstruction syndromes

| Characteristic | Kidney essence deficiency(n=87) | Phlegm and stasis obstructing the orifices  (n=93) | Heat toxin obstructing the channels(n=56) | χ²/H | *p* | *q* |  |  |  |
| --- | --- | --- | --- | --- | --- | --- | --- | --- | --- |
|  |  |  |  |  |  |  |  |  |  |
| Age/Years | 72.0 (69.0, 77.75) | 73.0 (65.0, 78.0) | 73.0 (68.0, 79.25) | 1.15 | 0.562 | 0.630 |  |  |  |
| Gender/Count(%) |  |  |  | 0.78 | 0.677 | 0.783 |  |  |  |
| Male | 27 (31.4) | 35 (37.63) | 19 (33.93) |  |  |  |  |  |  |
| Female | 59 (68.6) | 58 (62.37) | 37 (66.07) |  |  |  |  |  |  |
| Years of Education | 9.0 (6.5, 12.0) | 11.00 (9.0, 15.0) | 9.0 (9.0, 12.0) | 3.45 | 0.178 | 0.305 |  |  |  |
| Estimated symptom duration (years) | 3.5 (2.0, 5.0) | 3.00 (2.0, 5.0) | 5.0 (2.5, 8.0) | 11.58 | 0.003 | **0.009** |  |  |  |
| Hypertension(%) | 30 (34.48) | 37 (39.78) | 25 (44.64) | 1.52 | 0.468 | 0.748 |  |  |  |
| Diabetes(%) | 11 (12.79) | 19 (20.88) | 16 (28.57) | 5.45 | 0.065 | 0.262 |  |  |  |
| Heart Disease(%) | 7 (8.24) | 17 (18.28) | 4 (7.14) | 5.88 | 0.053 | 0.262 |  |  |  |
| Hyperlipidemia(%) | 12 (13.79) | 20 (21.98) | 15 (26.79) | 3.92 | 0.141 | 0.377 |  |  |  |
| GDS-30 | 3.0 (1.0, 8.0) | 3.0 (1.0, 7.5) | 6.5 (1.75, 12.5) | 2.37 | 0.306 | 0.409 |  |  |  |
| Smoking(%) | 11 (12.64) | 16 (17.20) | 8 (14.29) | 0.76 | 0.685 | 0.783 |  |  |  |
| Alcohol Consumption(%) | 12 (13.79) | 14 (15.22) | 10 (17.86) | 0.43 | 0.805 | 0.805 |  |  |  |
| Family History of Dementia | 16 (18.60) | 25 (26.88) | 11 (20.37) | 1.92 | 0.383 | 0.748 |  |  |  |
| MMSE | 15.0 (9.0, 21.0) | 13.0 (7.0, 20.0) | 5.0 (0.75, 16.0) | 17.73 | <0.001 | **<0.001** |  |  |  |
| MoCA | 11.0 (5.0, 15.5) | 7.5 (3.0, 14.0) | 4.0 (1.0, 11.0) | 18.75 | <0.001 | **<0.001** |  |  |  |
| ADL | 23.0 (17.5, 33.0) | 25.0 (18.0, 36.0) | 39.0 (23.75, 47.25) | 18.81 | <0.001 | **<0.001** |  |  |  |
| CDR | 1.0 (0.75, 2.0) | 1.0 (0.5, 2.0) | 2.0 (1.0, 3.0) | 26.46 | <0.001 | **<0.001** |  |  |  |
