# Supplementary material for: Cross-sectional associations of Kidney deficiency, Phlegm-stasis, and Heat-toxin syndrome patterns with disease severity in Alzheimer’s disease: an exploratory study
Source: Front Aging Neurosci. 2026 Oct 16;18:1763273. doi: 10.3389/fnagi.2026.1763273 (PMC13719365; doi:10.3389/fnagi.2026.1763273)
Supplement: Supplementary file 4 [file Data_Sheet_4.docx]

**TABLE S4** Comparison of peripheral blood biomarkers among patients with kidney essence deficiency, phlegm-stasis obstruction, and heat-toxin obstruction syndromes

| Characteristic | Kidney essence deficiency | Phlegm and stasis obstructing the orifices | Heat toxin obstructing the channels | H | *p* | *q* |
| --- | --- | --- | --- | --- | --- | --- |
| GFAP | 13.42 (8.36, 23.00) | 12.11 (7.76, 17.51) | 13.43 (10.07, 24.11) | 3.50 | 0.174 | 0.305 |
| Nfl | 10.70 (3.65, 18.75) | 9.18 (4.64, 15.64) | 13.04 (6.07, 20.62) | 3.01 | 0.222 | 0.333 |
| p-tau181 | 4.20 (2.63, 7.24) | 4.03 (1.97, 6.30) | 3.91 (2.64, 5.94) | 1.10 | 0.577 | 0.630 |
| p-tau217 | 5.69 (2.32, 8.54) | 6.12 (1.79, 9.39) | 7.57 (3.11, 13.46) | 6.29 | 0.044 | 0.171 |
| Aβ42/40 | 0.33 (0.15, 0.79) | 0.32 (0.10, 0.61) | 0.31 (0.13, 0.66) | 0.39 | 0.825 | 0.825 |
