# Supplementary material for: Cross-sectional associations of Kidney deficiency, Phlegm-stasis, and Heat-toxin syndrome patterns with disease severity in Alzheimer’s disease: an exploratory study
Source: Front Aging Neurosci. 2026 Oct 16;18:1763273. doi: 10.3389/fnagi.2026.1763273 (PMC13719365; doi:10.3389/fnagi.2026.1763273)
Supplement: Supplementary file 5 [file Data_Sheet_5.docx]

**TABLE S5** ROC curve analysis for predictive diagnostic efficacy across different TCM syndrome types in AD

| Characteristic | Kidney deficiency | 95% CI | Phlegm stasis | 95% CI | Heat toxin | 95% CI |
| --- | --- | --- | --- | --- | --- | --- |
| MMSEscale | 0.595 | 0.522–0.668 | 0.546 | 0.472–0.621 | 0.683 | 0.596–0.770 |
| ADLscale | 0.59 | 0.518–0.666 | 0.554 | 0.480–0.629 | 0.69 | 0.608–0.772 |
| Estimated symptom duration | 0.53 | 0.456–0.605 | 0.581 | 0.507–0.656 | 0.647 | 0.561–0.734 |
| CDR | 0.633 | 0.564-0.701 | 0.471 | 0.398-0.544 | 0.709 | 0.63-0.787 |
