# Supplementary material for: Cross-sectional associations of Kidney deficiency, Phlegm-stasis, and Heat-toxin syndrome patterns with disease severity in Alzheimer’s disease: an exploratory study
Source: Front Aging Neurosci. 2026 Oct 16;18:1763273. doi: 10.3389/fnagi.2026.1763273 (PMC13719365; doi:10.3389/fnagi.2026.1763273)
Supplement: Supplementary file 6 [file Data_Sheet_6.docx]

Table S6-1 Comparison of baseline characteristics among patients with kidney essence deficiency, phlegm-stasis obstruction, and heat-toxin obstruction syndromes (after excluding CDR = 0.5)

| Characteristic | Sum (n=186) | Kidney essence deficiency (n=65) | Phlegm and stasis obstructing the orifices (n=69) | Heat toxin obstructing the channels (n=52) | *p* | *q* |
| --- | --- | --- | --- | --- | --- | --- |
| Age/years | 73 (68, 79) | 73.50 (69, 78) | 74 (65, 79) | 73 (68, 77.50) | 0.052 | 0.149 |
| Years of education | 9 (7, 12) | 9 (6, 12) | 9 (9, 15) | 9 (8.75, 12) | 0.655 | 0.858 |
| MMSE | 10 (4, 16) | 12 (7, 17) | 10 (6, 16) | 4 (0, 15.25) | 0.008 | 0.073 |
| MoCA | 6 (2, 11) | 7 (4, 12) | 5 (3, 9) | 3 (1, 11) | 0.011 | 0.073 |
| ADL | 32 (23, 43) | 30 (21, 38) | 32 (24, 41) | 41.50 (24.75, 48.25) | 0.008 | 0.073 |
| Estimated symptom duration (years) | 4 (2, 6) | 4 (2, 5) | 3.50 (2, 6) | 5 (3, 8) | 0.020 | 0.100 |
| GFAP | 12.87 (8.47, 20.66) | 14.23 (8.29, 23.38) | 12.30 (7.11, 17.40) | 14.41 (10.33, 24.56) | 0.123 | 0.273 |
| NFL | 10.91 (4.71, 17.41) | 11.17 (4.32, 18.69) | 9.43 (4.23, 15.64) | 12.91 (6.07, 20.62) | 0.247 | 0.412 |
| P-Tau181 | 4.20 (2.56, 6.79) | 4.50 (2.82, 7.21) | 4.25 (2.15, 6.43) | 3.91 (2.64, 5.94) | 0.662 | 0.858 |
| P-Tau217 | 6.87 (2.64, 10.06) | 6.84 (2.95, 8.93) | 6.30 (1.92, 9.39) | 7.40 (3.11, 12.72) | 0.238 | 0.412 |
| Aβ42/40 | 0.32 (0.12, 0.66) | 0.31 (0.15, 0.67) | 0.37 (0.10, 0.66) | 0.30 (0.12, 0.63) | 0.968 | 0.968 |
| GDS-30 | 3 (1, 9) | 3 (1, 7) | 3 (1, 7) | 6.50 (1.75, 12.50) | 0.200 | 0.400 |
| Gender/case (%) |  |  |  |  | 0.908 | 0.956 |
| Male | 66 (35.7) | 22 (34.4) | 26 (37.7) | 18 (34.6) |  |  |
| Female | 119 (64.3) | 42 (65.6) | 43 (62.3) | 34 (65.4) |  |  |
| Smoking (%) |  |  |  |  | 0.729 | 0.858 |
| 0 | 161 (86.6) | 58 (89.2) | 59 (85.5) | 44 (84.6) |  |  |
| 1 | 25 (13.4) | 7 (10.8) | 10 (14.5) | 8 (15.4) |  |  |
| Alcohol consumption (%) |  |  |  |  | 0.729 | 0.858 |
| 0 | 158 (84.9) | 57 (87.7) | 58 (84.1) | 43 (82.7) |  |  |
| 1 | 28 (15.1) | 8 (12.3) | 11 (15.9) | 9 (17.3) |  |  |
| Hypertension (%) |  |  |  |  | 0.515 | 0.792 |
| 0 | 119 (64) | 44 (67.7) | 45 (65.2) | 30 (57.7) |  |  |
| 1 | 67 (36) | 21 (32.3) | 24 (34.8) | 22 (42.3) |  |  |
| Heart disease (%) |  |  |  |  | 0.077 | 0.192 |
| 0 | 167 (90.3) | 61 (95.3) | 58 (84.1) | 48 (92.3) |  |  |
| 1 | 18 (9.7) | 3 (4.7) | 11 (15.9) | 4 (7.7) |  |  |
| Diabetes (%) |  |  |  |  | 0.046 | 0.149 |
| 0 | 149 (81) | 58 (89.2) | 54 (80.6) | 37 (71.2) |  |  |
| 1 | 35 (19) | 7 (10.8) | 13 (19.4) | 15 (28.8) |  |  |
| Hyperlipidemia (%) |  |  |  |  | 0.042 | 0.149 |
| 0 | 153 (82.7) | 59 (90.8) | 56 (82.4) | 38 (73.1) |  |  |
| 1 | 32 (17.3) | 6 (9.2) | 12 (17.6) | 14 (26.9) |  |  |
| Family history of dementia |  |  |  |  | 0.813 | 0.903 |
| 0 | 140 (76.5) | 50 (78.1) | 51 (73.9) | 39 (78) |  |  |
| 1 | 43 (23.5) | 14 (21.9) | 18 (26.1) | 11 (22) |  |  |
| Note: Continuous variables are expressed as medians (P25, P75); categorical variables are expressed as counts (percentages). P-value: Continuous variables were analyzed using the Kruskal–Wallis test; categorical variables were analyzed using the χ² test. P-values were adjusted for FDR correction. | | | | | | |

**Table S6-2: Comparison of baseline characteristics across Western medicine diagnostic groups (after excluding CDR = 0.5)**

| Characteristic | Total (n=186) | Early-stage (n=77) | Moderate (n=66) | Severe (n=43) | *p* | *q* |
| --- | --- | --- | --- | --- | --- | --- |
| Age/years | 73 (68, 79) | 75 (70, 80) | 73 (65, 78) | 72 (66, 76) | 0.052 | 0.131 |
| Years of education | 9 (7, 12) | 9 (8, 12) | 9 (6, 12) | 9 (7.50, 12) | 0.655 | 0.928 |
| MMSE | 10 (4, 16) | 17 (13, 19) | 9 (6, 12) | 1 (0, 3.50) | <0.001 | <0.001 |
| MoCA | 6 (2, 11) | 12 (9, 14) | 4 (3, 7) | 1 (1, 1.75) | <0.001 | <0.001 |
| ADL | 32 (23, 43) | 23 (19, 28) | 35.50 (30, 40.50) | 47 (43, 51) | <0.001 | <0.001 |
| Estimated symptom duration (years) | 4 (2, 6) | 3 (2, 4) | 4 (2, 5) | 6 (4.03, 8) | <0.001 | <0.001 |
| GFAP | 12.87 (8.47, 20.66) | 11.23 (6.91, 17.89) | 13 (9.30, 21.88) | 17.40 (9.71, 29.24) | 0.015 | 0.061 |
| NFL | 10.91 (4.71, 17.41) | 7.99 (3.59, 15.23) | 11.18 (5.38, 17.57) | 13.55 (6.61, 23.93) | 0.037 | 0.106 |
| P-Tau181 | 4.20 (2.56, 6.79) | 4.19 (2.65, 6.14) | 4.24 (2.51, 6.38) | 4.03 (1.94, 7.38) | 0.995 | 0.995 |
| P-Tau217 | 6.87 (2.64, 10.06) | 6.85 (2.23, 9.53) | 6.90 (3.01, 9.31) | 6.88 (3.06, 11.28) | 0.833 | 0.980 |
| Aβ42/40 | 0.32 (0.12, 0.66) | 0.32 (0.09, 0.62) | 0.32 (0.15, 0.89) | 0.35 (0.15, 0.75) | 0.543 | 0.836 |
| GDS-30 | 3 (1, 9) | 3.50 (1.75, 8) | 3 (1, 10.50) | 2 (0, 7) | 0.245 | 0.545 |
| Gender/case (%) |  |  |  |  | 0.887 | 0.986 |
| Male | 66 (35.7) | 29 (37.7) | 22 (33.8) | 15 (34.9) |  |  |
| Female | 119 (64.3) | 48 (62.3) | 43 (66.2) | 28 (65.1) |  |  |
| Smoking (%) |  |  |  |  | 0.312 | 0.624 |
| 0 | 161 (86.6) | 64 (83.1) | 57 (86.4) | 40 (93) |  |  |
| 1 | 25 (13.4) | 13 (16.9) | 9 (13.6) | 3 (7) |  |  |
| Alcohol consumption (%) |  |  |  |  | 0.742 | 0.928 |
| 0 | 158 (84.9) | 64 (83.1) | 56 (84.8) | 38 (88.4) |  |  |
| 1 | 28 (15.1) | 13 (16.9) | 10 (15.2) | 5 (11.6) |  |  |
| Hypertension (%) |  |  |  |  | 0.458 | 0.763 |
| 0 | 119 (64) | 46 (59.7) | 46 (69.7) | 27 (62.8) |  |  |
| 1 | 67 (36) | 3 1(40.3) | 20 (30.3) | 16 (37.2) |  |  |
| Heart disease (%) |  |  |  |  | 0.940 | 0.989 |
| 0 | 167 (90.3) | 70 (90.9) | 58 (89.2) | 39 (90.7) |  |  |
| 1 | 18 (9.7) | 7 (9.1) | 7 (10.8) | 4 (9.3) |  |  |
| Diabetes (%) |  |  |  |  | 0.026 | 0.087 |
| 0 | 149 (81) | 62 (82.7) | 58 (87.9) | 29 (67.4) |  |  |
| 1 | 35 (19) | 13 (17.3) | 8 (12.1) | 14 (32.6) |  |  |
| Hyperlipidemia (%) |  |  |  |  | 0.713 | 0.928 |
| 0 | 153 (82.7) | 65 (84.4) | 55 (83.3) | 33 (78.6) |  |  |
| 1 | 32 (17.3) | 12 (15.6) | 11 (16.7) | 9 (21.4) |  |  |
| Family history of dementia |  |  |  |  | 0.414 | 0.753 |
| 0 | 140 (76.5) | 54 (72) | 53 (81.5) | 33 (76.7) |  |  |
| 1 | 43 (23.5) | 21 (28) | 12 (18.5) | 10 (23.3) |  |  |
| Note: Continuous variables are expressed as medians (P25, P75); categorical variables are expressed as counts (percentages). P-value: Continuous variables were analyzed using the Kruskal–Wallis test; categorical variables were analyzed using the χ² test. P-values were adjusted for FDR correction. | | | | | | |

Table S6-3: Correlation analysis between different dementia severity levels and various indicators (after excluding cases with CDR=0.5; n=185)

| Variable | *r* | *95% CI* | *p* |
| --- | --- | --- | --- |
| MMSE | -0.766 | -0.827~ -0.689 | <0.001 |
| MoCA | -0.771 | -0.834~ -0.697 | <0.001 |
| ADL | 0.716 | 0.628~ 0.797 | <0.001 |
| Estimated symptom duration | 0.362 | 0.226~ 0.490 | <0.001 |
| GFAP | 0.212 | 0.069~ 0.336 | 0.004 |
| Note: Spearman's rank correlation analysis was employed. A \|r\| ≥ 0.6 indicates a strong correlation; 0.4 ≤ \|r\| <0.6 indicates a moderate correlation; and \|r\| <0.4 indicates a weak correlation. The dependent variable is the severity of dementia (1 = mild, 2 = moderate, 3 = severe). | | | |

Table S6-4: Multiple logistic regression analysis of risk factors across different disease stages in Table AD (after excluding cases with CDR=0.5; n=185)

| Variable | Moderate AD vs Early-stage AD | | | Severe AD vs Early-stage AD | | |
| --- | --- | --- | --- | --- | --- | --- |
|  | β | *p* | OR (95% CI) | β | *p* | OR (95% CI) |
| MMSE | -0.146 | <0.001 | 0.86 (0.79–0.94) | -0.682 | <0.001 | 0.50 (0.37–0.69) |
| ADL | 0.114 | <0.001 | 1.12 (1.05–1.19) | 0.242 | <0.001 | 1.27 (1.14–1.42) |
| Phlegm stagnation | 0.230 | <0.001 | 1.26 (1.12–1.42) | 0.225 | 0.032 | 1.25 (1.02–1.54) |
| Heat toxin | 0.181 | 0.004 | 1.20 (1.06–1.35) | 0.404 | <0.001 | 1.50 (1.22–1.84) |
| Kidney deficiency | 0.028 | 0.522 | 1.03 (0.94–1.12) | -0.117 | 0.287 | 0.89 (0.72–1.10) |
| Estimated symptom duration | 0.010 | 0.842 | 1.01 (0.92–1.11) | -0.042 | 0.528 | 0.96 (0.84–1.09) |
| GFAP | 0.013 | 0.188 | 1.01 (0.99–1.03) | 0.029 | 0.158 | 1.03 (0.99–1.07) |
| Note: A multinomial logistic regression model was fitted with AD stage (early-stage vs. moderate vs. severe; early-stage as reference) as the outcome and MMSE, ADL, estimated symptom duration, GFAP, kidney essence deficiency, phlegm and stasis, and heat toxin as predictors; MoCA was excluded due to collinearity with MMSE. Given violation of the proportional odds assumption (omnibus LR test: χ² = 15.79, df = 7, *p* = 0.027), a multinomial rather than ordinal model was adopted. The overall model test was significant (χ² = 237.775, df = 14, *p* < 0.001). Model fit statistics: AIC = 192.76; McFadden's pseudo R² = 0.597. | | | | | | |
