# Supplementary material for: Cross-sectional associations of Kidney deficiency, Phlegm-stasis, and Heat-toxin syndrome patterns with disease severity in Alzheimer’s disease: an exploratory study
Source: Front Aging Neurosci. 2026 Oct 16;18:1763273. doi: 10.3389/fnagi.2026.1763273 (PMC13719365; doi:10.3389/fnagi.2026.1763273)
Supplement: Supplementary file 7 [file Data_Sheet_7.docx]

**TCM Scoring Scale**

| **Kidney Essence Deficiency** | **Phlegm-Stasis Blocking Orifices** | **Heat-Toxin Blocking Collaterals** |
| --- | --- | --- |
| Memory  Recent amnesia (2 points)  Remote amnesia (3 points) | Memory  Recent amnesia (2 points)  Remote amnesia (3 points) | Memory  Recent amnesia (2 points)  Remote amnesia (3 points) |
| Loin and Leg  Loin soreness (3 points)  Loin and leg soreness and weakness (4 points)  Loin and spinal soreness (5 points) | Mental State  Indifferent expression or oligophasia (2 points)  Slow response or somnolence (3 points) | Mental State  Irritability (2 points)  Irritability with insomnia (3 points)  Delirium (4 points) |
| Night Sweats  Present (1 point) | Pain  Fixed pain (2 points)  Stabbing pain (3 points) | Facial Appearance and Fever  Facial flushing or fever (1 point)  Facial flushing with bloodshot eyes accompanied by fever (2 points) |
| Ear  Tinnitus like cicada chirping (2 points)  Deafness (3 points)  Atrophic ear helix (4 points) | Head  Dizziness (1 point)  Heavy dizziness (2 points) | Breathing  Loud and rough breathing or shortness of breath (1 point)  Foul-smelling breath or halitosis (2 points) |
| Hair and Teeth  Hair loss (2 points)  Loose teeth (3 points)  Tooth loss (4 points) | Phlegm  Profuse and sticky phlegm (2 points)  Snoring with phlegm rales (3 points) | Drinking and Urine  Dry and red lips or yellow urine (2 points)  Fond of drinking with scanty, dark and hot urine (3 points)  Oral ulcers or painful hemorrhoids (4 points) |
| Urine and Sexual Function  Decreased sexual function (2 points)  Impotence (3 points)  Dribbling urine after micturition (5 points)  Frequent nocturia (6 points) | Facial Complexion  Cyanosis of lower face or purple and dark lips (2 points)  Dark complexion and purple and dark lips (3 points) | Appetite  Decreased appetite (2 points)  Food intake reduced by half (3 points) |
| Tongue Nature  Pale tongue (1 point),  Red tip of tongue (2 points)  Thin, red and dry tongue (3 points)  Thin, red, dry and cracked tongue (4 points) | Tongue Nature  Purple and dark tongue (1 point)  Purple and dark tongue with ecchymoses and petechiae (2 points)  Cyanotic tongue (3 points) | Stool  Dry stool (2 points)  No defecation for 2 or 3 days (3 points)  No defecation for 4 or 5 days (4 points)  No defecation for 6 days or more (5 points) |
| Tongue Coating  Thin, white and scanty coating (1 point)  No coating (2 points) | Tongue Body  Enlarged tongue (2 points)  Enlarged tongue with multiple tooth marks (3 points) | Tongue Nature  Red tongue (1 point)  Deep red tongue (2 points) |
| Pulse Condition  Thin and weak pulse (1 point) | Tongue Coating  Sticky or slippery coating (2 points)  Thick sticky or turbid sticky coating (3 points) | Tongue Coating  Yellow or thick yellow sticky coating (1 point)  Grayish-black and dry coating (2 points) |
|  | Pulse Condition  Slippery or soft pulse (2 points)  Deep, string-like and thin pulse (3 points)  Astringent or irregularly intermittent pulse (4 points) | Pulse Condition  Slippery pulse (2 points)  Rapid, large and forceful pulse, or string-like and rapid pulse, or slippery and rapid pulse (3 points) |
